# Third-world realities in a first-world setting: A study of the HIV/AIDS-related conditions and risk behaviors of sex trade workers in Saskatoon, Saskatchewan, Canada

**DOI:** 10.1080/17290376.2016.1229213

**Published:** 2016-09-11

**Authors:** Yelena Bird, Mark Lemstra, Marla Rogers, John Moraros

**Affiliations:** ^a^ MD, PhD, MPH, is an Assistant Professor at the School of Public Health, University of Saskatchewan, Saskatchewan, Canada; ^b^ DrPH, PhD, is an Adjunct Professor at the School of Public Health, University of Saskatchewan, Saskatchewan, Canada; ^c^ BA, MPA, is a Researcher at the College of Medicine, University of Saskatchewan, Saskatchewan, Canada; ^d^ MD, PhD, MPH, is an Associate Professor at the School of Public Health, University of Saskatchewan, Saskatchewan, Canada

**Keywords:** sex trade workers, risk factors, HIV/AIDS, aboriginal populations, Canada, les travailleurs du sexe, facteurs de risque, VIH / SIDA, populations autochtones, Canada

## Abstract

The transmission and prevalence of Human Immunodeficiency Virus (HIV) among those employed as sex trade workers (STW) is a major public health concern. The present study describes the self-reported responses of 340 STW, at-risk for contracting HIV. The participants were recruited by selective targeting between 2009 and 2010 from within the Saskatoon Health Region (SHR), Saskatchewan, Canada. As of 2012, the SHR has the highest incidence rate of positive test reports for HIV in Canada, at more than three times the national average (17.0 vs. 5.9 per 100,000 people). Additionally, the epidemiology of HIV/AIDS in the SHR is different from that seen elsewhere in Canada (still mostly men having sex with men and Caucasians), with its new HIV cases predominantly associated with injection drug use and Aboriginal cultural status. The purpose of this study was to (a) describe the demographic and socio-economic characteristics of the STW in the SHR, (b) identify their significant life events, self-reported problems, knowledge, attitudes, behaviors, self-efficacy, and barriers regarding HIV, and (c) determine the significant independent risk indicators for STW self-reporting a chance of greater than 50% of becoming infected with HIV/AIDS. The majority of the study participants were females, who were never married, of Aboriginal descent, without a high school diploma, and had an annual income of less than $10,000. Using multivariate regression analysis, four significant independent risk indicators were associated with STW reporting a greater that 50% chance of acquiring HIV/AIDS, including experiencing sexual assault as a child, injecting drugs in the past four weeks, being homeless, and a previous Chlamydia diagnosis. These findings provide important evidence of the essential sexual and drug-related vulnerabilities associated with the risk of HIV infection among STW and offer insight into the design and implementation of effective and culturally sensitive public health intervention and prevention efforts. To be most effective, it is recommended that such intervention and prevention initiatives: (1) use specifically tailored community-based outreach to high risk STW who are drug users and link them with appropriate drug treatment and HIV/AIDS prevention and treatment services, (2) provide free and confidential, routine HIV counseling and testing in substance abuse programs, and (3) build capacity among the local, Aboriginal NGOs so as to address with cultural sensitivity both the drug and HIV-related risk factors prevalent among this vulnerable population.

## Résumé

La transmission et la présence élevée du virus de l’immunodéficience humaine (VIH) parmi les personnes travaillant dans le commerce du sexe sont des problèmes de santé publique majeurs. La présente étude décrit les déclarations faites par 340 travailleurs du sexe à risque d’attraper le VIH. Les participants ont été recrutés par ciblage sélectif entre 2009 et 2010 dans la région sociosanitaire de Saskatoon, en Saskatchewan, au Canada. Depuis 2012, cette région sociosanitaire possède le taux d’incidence le plus élevé de résultats positifs aux tests de dépistage du VIH au Canada. Son taux est trois fois plus élevé que la moyenne nationale (17.0 par rapport à 5.9 pour 100 000 personnes). De plus, l’épidémiologie du VIH/SIDA dans cette région sociosanitaire est différente de celle observée ailleurs au Canada (où il s'agit encore d'une majorité d’hommes ayant des rapports sexuels avec des hommes et de personnes caucasiennes). Les nouveaux cas de VIH de cette région sociosanitaire sont essentiellement associés à l’utilisation de drogues par injection et à l’appartenance à la population autochtone. La présente étude avait pour objectif (a) de décrire les caractéristiques démographiques et socio-économiques des travailleurs du sexe dans la région sociosanitaire, (b) de cibler les événements importants de leur vie, les problèmes qu’ils ont signalés, leurs connaissances, leur vision des choses, leur comportement, leur efficacité personnelle et leurs barrières par rapport au VIH, et (c) de déterminer les indicateurs de risque importants pour les travailleurs du sexe déclarant un risque de plus de 50 % de contracter le VIH/SIDA. La majorité des participants à cette étude étaient des femmes d’origine autochtone n’ayant jamais été mariées, ne possédant pas de diplôme d’études secondaires et dont le revenu annuel était de moins de 10 000 $. En utilisant comme cadre une analyse de régression multivariée, quatre principaux indicateurs de risques ont été associés aux travailleurs du sexe déclarant un risque de plus de 50 % de contracter le VIH/SIDA, dont le fait d’avoir été victime d’agression sexuelle durant l’enfance, de s’être injecté de la drogue dans les quatre dernières semaines, d’être sans-abri, et d’avoir reçu un diagnostic antérieur de chlamydia. Ces conclusions constituent une preuve importante des vulnérabilités sexuelles et liées à la drogue associées au risque d’être infecté par le VIH pour les travailleurs du sexe et donnent un aperçu de la conception et de la mise en place d’interventions en matière de santé publique et d’initiatives de prévention efficaces et culturellement adaptées. Pour être le plus efficaces possible, les interventions et les initiatives de prévention devraient : 1) comprendre une aide sociale basée sur la communauté et spécialement conçue pour les travailleurs du sexe hautement à risque qui utilisent des drogues, qui permet de les référer à un traitement de la toxicomanie approprié et à des services de prévention et de traitement du VIH/SIDA, 2) fournir des services de conseil et de dépistage du VIH gratuits et confidentiels dans le cadre des programmes de traitement de la toxicomanie, et 3) donner les moyens aux ONG autochtones locales de réagir de façon culturellement appropriée aux facteurs de risques liés à la drogue et au VIH présents dans la population vulnérable.

## Introduction

In recent years, epidemiological studies in Canada have shown a progressive decline in the rates by which Human Immunodeficiency Virus (HIV) is spreading among the general population from an estimated 2800 new cases in 2011 to 2570 new cases in 2014 (Public Health Agency of Canada [PHAC] 2015). However, the national decreases in HIV rates, although significant in number, are not representative or generalizable to all provinces, cities, and sub-groups of the Canadian population (PHAC 2014a). In fact, they may be masking an expanding spread among certain neglected and vulnerable populations, who are most at risk to contract HIV and experience a disproportionately higher burden of disease. One such at-risk group is that of sex trade workers (STW) in the city of Saskatoon, Saskatchewan, Canada (PHAC 2014b).

STW are those who receive money, drugs, shelter, or goods in exchange for sexual services, either regularly or occasionally, and who may or may not consciously define those activities as income-generating (UNAIDS 2009). According to the Canadian Penal Code, the buying and selling of sexual services are legal. However, sex-trade-related activities (such as public communication for the purpose of prostitution, brothels, and procuring) are outlawed (Lowman 2004). This ambiguous and contradictory status creates a set of complex interrelationships that cloud and limit our understanding of the burden of HIV among STW in Canada (McCarthy, Benoit & Jansson 2012; Report of the Subcommittee on Solicitation Laws 2006).

The exact number of STW in Canada is unknown and information on this topic is rather limited. This is mainly a reflection of the fact that STW are poorly represented in national HIV surveillance systems and many wish to stay hidden due to fear of criminal prosecution and social stigmatization (Benoit, McCarthy & Jansson 2015). However, what is known is that sex work exists in all major Canadian cities, it is highly gendered (predominantly females) and racialized (mainly Aboriginal people) and plays a significant role in the spread of sexually transmitted infections (STI) and HIV/AIDS (Canadian Public Health Association 2014; Roy, Haley, LeClerc, Lemire, Boivin, Frappier, *et al.* 2000).

During the last decade, the highest incidence of positive HIV tests has been reported by the Saskatoon Health Region (SHR) in the province of Saskatchewan (PHAC 2014b). In 2012, the HIV rate in the SHR was nearly three times the national average (17.0 vs. 5.9 per 100,000 people) (Saskatoon Health Region 2015). Interestingly, the epidemiology of HIV/AIDS in the SHR is quite different from that seen elsewhere in Canada (still mostly men having sex with men and Caucasians), with its new HIV cases predominantly associated with injection drug use (IDU) and Aboriginal cultural status (Moraros, Falconer, Rogers & Lemstra 2012).

Although estimates vary widely by city and country, considerable overlap has been reported between STW and injection drug users (IDUs). These rates range from 14% and 22% respectively in two major border cities in Mexico (Strathdee, Lozada, Martinez, Vera, Rusch, Nguyen, *et al.* 2011), 51% among seven cities in the United States (Centers for Disease Control [CDC] 1987), 58% in China (Chen, Yin, Liang, Gong, Li, Poumerol, *et al*. 2005), and 71% in Scotland (McKeganey, Barnard, Leyland, Coote & Follet 1992). By comparison, 76.9% (133 of 173) of the positive HIV test reports in Saskatoon were associated with IDUs, which represents more than four times the national Canadian average (18.9%) (Saskatchewan Ministry of Health 2009).

This is a troubling development because just a decade ago, the rate of new HIV infections in the city of Saskatoon was practically identical to the national Canadian average (approximately 5 per 100,000 people) (PHAC 2004). However since that time, it has soared to unprecedented heights (95 per 100,000 people) (PHAC 2014b). These findings may be attributed in large part to the documented links between the HIV epidemic with IDU and the sex trade (Craib, Spittal, Wood, Laliberte, Hogg, Li, *et al.* 2003; El-Bassel, Witte, Wada, Gilbert & Wallace 2001; Moraros *et al.* 2012; Simoni, Sehgal & Walters 2004).

In Canada, there has been a limited amount of research conducted among IDUs and their possible links to the sex trade industry (Shannon, Kerr, Bright, Gibson & Tyndall 2008; Weber, Boivin, Blais, Haley & Roy 2002). A recent study in the SHR reported that high risk IDUs were more often engaged in giving sex to get drugs and giving drugs to get sex (Moraros *et al.* 2012). This is an important finding as transmission of HIV may be potentiated in settings where there are high proportions of STW who inject drugs because they can acquire and transmit HIV through both the use of contaminated syringes and unsafe sexual practices.

The present study endeavors to explore the complex relationships of the sex trade in the city of Saskatoon, Saskatchewan, Canada in several meaningful ways. Currently, the demographic and socio-economic characteristics of STW in Saskatoon have not been reported. Additionally, their significant life events, self-reported problems, knowledge, attitudes, behaviors, self-efficacy, and barriers regarding HIV have not been identified. Finally, the significant independent risk indicators for STW self-reporting a chance of greater than 50% of becoming HIV/AIDS positive have not been determined. Consequently, most STW living with HIV or at-risk to develop HIV face significant challenges in accessing much needed prevention, care, and treatment services.

## Methods

### Study setting

The present study was conducted in Saskatoon, Saskatchewan, Canada. Saskatoon is the largest city in the province with an estimated population of approximately 257,300 as of 1 July 2012 and one of the fastest growing cities in Canada. Approximately one-fifth of its population consists of youth under the age of 15 years (19%), while those over 65 years old constitute 13% of the population. The median age of Saskatoon residents is 35.5 years, which is five years younger than the national median age in Canada (40.6 years old). Nearly 10% of the people in the city identify themselves as Aboriginal (Statistics Canada 2011).

According to Statistics Canada’s 2011 National Household Survey (a voluntary sampling of the population), the median income of Saskatoon families was $73,519 CAD in 2010. However, the data demonstrated a significant disparity with 21% of the people in the city earning $12,025 CAD or less annually, while the top 5% earned more than $100,000 CAD. Nearly half of the population (44%) earned $27,815 a year or less annually and close to one-in-five children in Saskatoon lived in poverty (18.5%) (Statistics Canada 2011).

### Study design & participants

This was a quantitative cross-sectional descriptive study. From the months of September 2009 to April 2010, 340 STW at-risk for contracting HIV were recruited to participate through targeted sampling in the city of Saskatoon, Saskatchewan, Canada. Only adults aged 18 years or older, who gave both written and informed consent were permitted to participate in the study. Respondents were given a $20 honorarium. Ethics approval was obtained from the University of Saskatchewan Behavioral Research Ethics Board (BEH# 08–53).

### Survey tools

The UNAIDS (2002) guidelines were used in this study to help define and identify current STW. Study participants were asked two questions. First, respondents were asked if they had ever given sex (tricked) to get money within the last six months. Second, respondents were asked if they have ever given sex (tricked) to get drugs within the last six months. A current STW was defined as someone who answered yes to either question. The Spearman correlations for these questions were *r* = .72 and *r* = .78, respectively (Johnson, Fisher, Montoya, Booth, Rhodes, Andersen, *et al.* 2000).

### Sociodemographic information

Demographic and socio-economic status information was collected using questions taken from the Risk Behaviors Assessment Questionnaire created by the National Institute on Drug Abuse. This questionnaire was appropriate for use in Canada as it has been previously used and validated in both the Vancouver Injection Drug User Study (VIDUS) and the At Risk Youth Survey (ARYS) (British Columbia Center for Excellence in HIV/AIDS 2006; Craib, Spittal, Wood, Laliberte, Hogg, Li, *et al.* 2003; Wood, Montaner, Li, Zhang, Barney, Strathdee, *et al.* 2008). The reliability of the survey in determining the self-reported status of the participants was deemed to be satisfactory (kappa coefficients = .78 to 1.00) (Johnson, Fisher & Reynolds 1999).

### HIV/AIDS-related knowledge & behaviors

Participant’s HIV/AIDS-related knowledge was measured using a series of 28 questions taken from the Health and Relationships Survey, AIDS Preventive Behaviors. This survey has been reported to have a strong reliability (alpha ranging from .68 to .72) (Koopman & Reid 1998). Behaviors associated with increased risk of HIV infection (such as trading sex, number of sex partners, frequency of injection, and sharing of injecting equipment) were measured using the Risk Behaviors Assessment Questionnaire mentioned previously.

### Statistical analysis

All data analyses were conducted using the SPSS statistical software package. Frequencies, means, and standard deviations were used to describe the participants and their responses on the survey instruments. Hierarchal well-formulated, binary logistic regression was used to determine the association between the outcome variable (participant’s self-reporting their chance of getting HIV/AIDS as below or above 50%) and various covariates. The original question asked respondents to self-report their risk as no chance (0%), some chance (25%), half chance (50%), high chance (75%), or sure chance (100%). In order to compute logistic regression, the answers were collapsed into only two categories (below or above 50%). The unadjusted effect of each covariate was determined and then entered one step at a time based on changes in the −2 log likelihood and the Wald Test (Rothman & Greenland 1998). The final results are presented as adjusted odds ratios with 95% confidence intervals. Statistical significance was set at *p*-value < .05.

## Results

### Sociodemographic characteristics

There were 340 individuals who participated in this study and self-identified as being STW. Their demographic and socio-economic characteristics are presented in Table 1. The majority of the study participants were females (69.9%), who were never married (67.4%), of Aboriginal descent (89.7%), without a high school diploma (60.0%), and had an annual income of less than $10,000 (75.8%) (Table 1).

**Table 1.** Demographic and socio-economic status of STW in Saskatoon.

	*N* = 340 Percentage
*Gender*	
Male	30.1
Female	69.9
*Age*	
18–29	30.1
30–39	36.3
40–69	33.6
*Marital status*	
Never married	57.4
Married	4.4
Common law	24.4
Separated, divorced, widowed, or other	13.8
*Cultural status*	
Caucasian	9.4
Aboriginal (First Nations, Métis or Inuit)	89.7
Other	0.9
*Highest level of school completed*	
Eight grade or less	18.5
Grade 9-12 but no high school graduation	60.0
Completed high school	13.4
Trade or technical training	4.2
College certificate	3.9
University degree	0.0
*Homeless*	
Yes	40.2
No	59.8
*If housed, where are they living now?*	
Own home or apartment	40.2
Someone else’s home or apartment	41.4
Shelter	7.1
Other	11.3
*Employment*	
Unemployed	44.9
Working full-time	2.1
Working part-time	4.5
Disabled, not able to work	29.9
Other	18.6
*Sources of income*	
Paid job or salary	6.6
Social security or disability	41.5
Sell or trade goods, barter	6.6
Other	45.3
*Annual household income*	
$0–$9999	75.8
$10,000–$19,999	17.0
$20,000–$29,999	3.5
$30,000–$39,999	2.8
More than $40,000	0.9

### Significant life events

There were several significant life events self-reported by the STW. Among the most frequently mentioned were having suffered physical assault or abuse in their adult life by either their partner or someone other than their partner (76.1% and 74.7% respectively), being the victim of physical assault or sexual abuse as a child (76.2% and 69.1% respectively), seeing people hitting or harming one another in their family while growing up (86.8%), and having a parent or grandparent who attended Residential School (70.8%). Significant life events are presented in Table 2.

**Table 2.** Significant life events of STW in Saskatoon.

*Experienced*:	*N* = 340 Percentage
Physical assault or abuse in their adult life by their partner	76.1
Physical assault or abuse in their adult life by someone other than their partner	74.7
Physical assault or abuse as a child	76.2
Seeing people hitting or harming one another in their family while growing up	86.8
Sexual assault in their adult life	57.9
Sexual assault as a child	69.1
Seeing someone physically assaulted or abused	86.7
Seeing someone seriously injured or violently killed	67.7
Losing a child through death	34.8
Death or permanent separation from a parent or someone who was like a parent before 18 years of age	56.8
Death of a spouse, partner, or loved one as an adult	62.2
Attended a Residential School	37.0
Had a parent or grandparent who attended Residential School	70.8

### HIV/AIDS-related problems, knowledge & attitudes

Most STW reported HIV as being a serious problem in their community (91.6%). Many attributed this problem to substance/drug abuse and unprotected sexual practices (62.9% and 46.8%, respectively). Insofar as HIV/AIDS knowledge was concerned, they were more likely to incorrectly answer the following HIV-related knowledge statements, ‘people (can) get other diseases because of AIDS,’ ‘coughing and sneezing spread HIV’, and ‘all pregnant women with HIV will have babies born with HIV.’ Additionally, their attitudes were also a concern with nearly one-third of them either somewhat or strongly agreeing to being ‘tired of always having to make sure I practice safe sex’ and being ‘tired of always having to make sure not to share needles with others’ (29.1% and 32.7% respectively) (Table 3).

**Table 3.** Self-reported problems, knowledge, attitudes, behaviors, self-efficacy, and barriers.

	*N* = 340 Percentage
*Problem*	
Is HIV a problem in your community, yes	91.6
Why? Substance/drug abuse	62.9
Unprotected sex	46.8
Lack of knowledge	32.1
Lack of perceived risk	21.2
*Knowledge*	
(Only scores with greater than 10% wrong answers listed)	Percentage wrong
Most people who develop AIDS eventually recover	12.9
You can’t get HIV if you only have one partner	17.6
People get other diseases because of AIDS	41.8
Using condoms will lessen the chance of getting AIDS	15.5
It is safe to have intercourse without a condom with a person who shoots drugs as long as you do not shoot drugs.	10.0
The virus from AIDS can be passed by an infected person even though the infected person isn’t visibly sick	19.1
Using drugs/alcohol makes it more likely that you will have unsafe sex	22.1
Coughing and sneezing spread HIV	32.4
A woman can get HIV if she has anal sex with a man	16.6
All pregnant women with HIV will have babies born with HIV	39.4
People infected with HIV quickly show serious signs of being infected	21.0
There is a vaccine that stops adults from getting HIV	16.5
A woman cannot get HIV if she has sex during her period	16.4
Having sex with more than one partner increases the chances of being infected with HIV	13.1
A person can get HIV from oral sex	23.0
*Attitudes*	Percentage
I am tired of always having to make sure I practice safe sex	
Somewhat agree or strongly agree	32.7
It’s too much of a hassle to practice safe sex all of the time	
Somewhat agree or strongly agree	21.4
I find it hard to practice safe sex all of the time	
Somewhat agree or strongly agree	22.5
I am tired of always having to make sure not to share needles with others	
Somewhat agree or strongly agree	29.1
It’s too much of a hassle to use clean needles all the time when I inject	
Somewhat agree or strongly agree	19.2
I find it hard to use clean needles all the time when I inject	
Somewhat agree or strongly agree	22.5
*Behaviors*	Mean (SD)
In the past month, number of times had vaginal, oral or anal sex	8.10 (9.63)
In the past month, number of people had vaginal, oral or anal sex with	2.60 (5.14)
In the past month, number of times injected with drugs	3.70 (6.14)
	Percentage
Have injected a drug in the past four weeks, yes	71.5
Of those who injected, how often have you shared injecting equipment?	
Sometimes or frequently	65.5
*Self-Efficacy*	
I can tell a sex partner that I do not want to inject drugs	
Greater than 50% chance	54.9
I can resist injecting if a sex partner offers to inject me	
Greater than 50% chance	46.6
I can talk about safe sex with a partner	
Greater than 50% chance	60.8
I can refuse to have sex with someone	
Greater than 50% chance	62.4
I can convince a sex partner to use a condom	
Greater than 50% chance	70.6
I can prevent a partner from having anal sex with me	
Greater than 50% chance	68.9
I can ask a sex partner about their previous sexual partners	
Greater than 50% chance	63.0
*Medical*	Percentage
Depressed mood (measured by CES-D)	85.6
Previously diagnosed by a doctor or nurse with:	
Hepatitis B	46.5
Gonorrhoea	42.3
Syphillis	27.1
Genital warts	30.1
Chlamydia	53.8
Herpes	24.5
Previously tested for HIV/AIDS, yes	90.3
*Barriers*	Percentage
Which of the following are problems:	
Long distance to medical facilities and personnel	
Somewhat/ major problem	38.8
Medical personnel who decline to provide direct care	
Somewhat/ major problem	31.5
Lack of healthcare practitioners who are adequately trained	
Somewhat/ major problem	38.5
Lack of mental health counselors who can address mental health	
Somewhat/ major problem	38.5
Lack of psychological support	
Somewhat/ major problem	46.2
Community stigma towards persons living with HIV/AIDS	
Somewhat/ major problem	61.9
Lack of employment opportunities	
Somewhat/ major problem	66.1
Lack of financial resources	
Somewhat/major problem	60.6
Lack of adequate and affordable housing	
Somewhat/ major problem	73.0
Unable to get into a drug treatment or detox program, Yes	42.6

### Self-reported behaviors

Equally revealing were the behaviors of the STW over the past month. They reported an average of 8.1 vaginal, oral, or anal sexual episodes, with 2.6 different sexual partners, while being under the influence of injectable drugs in 3.7 of those occasions. Nearly three-fourths (71.5%) admitted to IDU during the past four weeks, while of those who injected, 65.5% reported either sometimes or frequently sharing injecting equipment. A large proportion reported being unable to get into a drug treatment or detox program (42.6%) (Table 3).

### Self-efficacy & barriers

Regarding the self-efficacy of the STW participating in the study, it is interesting to note that all relevant questions demonstrated a greater than 50% chance of agreement with the sole exception of the question on whether ‘I can resist injecting if a sex partner offers to inject me’ at 46.6%. Additionally, most STW surveyed were found to be of depressed mood (as measured by CES-D) at 85.6%. The majority had been previously tested for HIV/AIDS at 90.3% and diagnosed with Chlamydia infection by a doctor or nurse in the past at 53.8%. Finally, the major barriers identified by the STW were mainly the ‘community stigma towards persons living with HIV/AIDS’ at 61.9%, ‘lack of employment opportunities’ at 66.1%, ‘lack of financial resources’ at 60.6% and ‘lack of adequate and affordable housing’ at 73.0% (Table 3).

### Regression analysis

After multivariate regression analysis, the four statistically significant independent risk indicators for STW self-reporting a chance of greater than 50% of getting HIV/AIDS included experiencing a sexual assault as a child (odds ratio 2.04, 95% confidence interval 1.11–3.73), injecting drugs in the past four weeks (odds ratio 1.41, 95% confidence interval 1.06–1.68), being homeless (odds ratio 1.29, 95% confidence interval 1.09–1.59), and having been diagnosed with a Chlamydia infection by a doctor or nurse in the past (odds ratio 1.26, 95% confidence interval 1.08–1.47) (Table 4).

**Table 4.** Independent risk indicators of self-reporting chance of getting HIV/AIDS as greater than 50%.

Independent variables	OR	95% Confidence interval	*P*-value
Sexual assault as a child, yes	2.04	1.11–3.73	.021
Injected drug in past four weeks, yes	1.41	1.06–1.68	.040
Consider self to be homeless, yes	1.29	1.09–1.59	.039
Diagnosed with Chlamydia in past, yes	1.26	1.08–1.47	.003
Reference categories:			
No sexual assault as a child			
Did not inject drug in past four weeks			
Does not consider self to be homeless			
Has not been diagnosed by doctor or nurse in past for Chlamydia			
Hosmer–Lemeshow test = .246			
Nagelkerke *R* Squared = .109			

## Discussion

The high rate of HIV-positive test reports in Saskatoon, Saskatchewan, Canada is a major public health concern. The present study into the HIV/AIDS-related risks among STW in the region uncovered four important findings. First, STW reported experiencing a vast array of significant life events which place them at increased risk for contracting HIV and revealed a strong association between childhood physical and sexual assault and future sex trade work. Second, STW reported high rates of IDU and perceived factors associated with their injection behaviors to be more closely associated with the risk of HIV infection than lack of HIV knowledge or unsafe sexual practices. Third, a majority of the STW were Aboriginal and most reported being depressed. Finally, the significant independent risk indicators for a STW self-reporting a chance of greater than 50% of becoming HIV/AIDS positive were identified. These findings have salient implications in the design and implementation of effective public health interventions and emphasize the importance of culturally appropriate strategies, which are critical in the interruption of HIV transmission behaviors among STW.

It has been widely reported that STW who exchange sex for money or drugs are significantly more likely to have been the victims of violence and sexual assault in adulthood (Argento, Muldoon, Duff, Simo, Deering & Shannon 2014; Muldoon, Deering, Feng, Shoveller & Shannon 2015; Phillips, Walsh, Bullion, Reid, Bacon & Okoro 2014). Unfortunately in Canada, the victimization of STW has reached a critical precipice in many major cities such as Calgary, Winnipeg, and Vancouver (Amnesty International 2004; Mehrabadi, Craib, Patterson, Adam, Moniruzzaman, Ward-Burkitt, *et al.* 2008; Shannon, Kerr, Allinott, Chettiar, Shoveller & Tyndall 2008) but very little is known about their experiences in Saskatoon. The present study helps us to contribute to our body of knowledge in this area by reporting that the majority of STW in Saskatoon were Aboriginal (89.7%) and had suffered traumatic experiences during childhood and adulthood by being the victims of both physical (76.2% and 75.4.%, respectively) and sexual abuse (69.1% and 57.9%, respectively). This evidence further supports the premise that violence decreases the ability of STW to successfully negotiate safe sex practices (Kerr, Shannon, Ti, Strathdee, Hayashi, Nguyen, *et al.* 2016; Long, DeBeck, Feng, Montaner, Wood & Kerr 2014; Rekart 2005) and highlights the urgent need for harm-reduction initiatives specifically focused to address violence in both working and intimate relationships for this vulnerable population.

Over the last decade, the upsurge in IDU practice in Saskatoon helps explain in part the concomitant increase in HIV infection rates observed in the city (PHAC 2009, 2014b). A previous study in Saskatoon reported that the main characteristics of high risk IDUs were greatly reflective of their injecting behaviors and practices (Moraros *et al.* 2012). Similarly, the STW participating in the present study reported high rates of IDU. They also perceived factors associated with their injection behaviors such as drug-dependence, injecting with others, and sharing injecting equipment to be more closely associated with their risk of HIV infection than lack of HIV knowledge or unsafe sexual practices. However, despite the seriousness of their drug-use issue, almost half of the STW reported being unable to get into a drug treatment or detox program. Our findings help emphasize the need to expand drug treatment services and the importance of making clean needles and syringes easily accessible to STW who inject drugs, if we are to successfully combat the rapid rise of HIV in the region.

In Canada and specifically in the city of Saskatoon, people of Aboriginal ancestry are over-represented both in the sex trade and the HIV epidemic (Lemstra, Rogers, Thompson, Moraros & Buckingham 2012; PHAC 2004). The unique vulnerabilities facing STW of Aboriginal ancestry have been well documented. These mainly stem from the multigenerational effects of continued discrimination, social dislocation, inequalities/inequities in health, abject poverty, and even the lingering effects of the residential school system (Farley, Lynne & Cotton 2005; Lemstra, Rogers, Thompson, Moraros & Buckingham 2012; PHAC 2009). Therefore, there is growing evidence which supports the need for public health strategies which require mental health services, community empowerment, and indigenous healing to help mitigate the matrix of lifetime trauma/abuse, IDU, and HIV vulnerability (BC Aboriginal HIV/AIDS Task Force 2004; Mehrabadi, Craib, Patterson, Adam, Moniruzzaman, Ward-Burkitt, *et al.* 2008; Simoni *et al.* 2004).

It has been previously reported that lack of attention to the social determinants of health greatly influence the development of health inequalities/inequities and disproportionately place vulnerable and marginalized populations such as STW and Aboriginal people at increased risk for poor health outcomes (Coleman, Tate, Gaddist & White 2016; Juan-Martínez & Castillo-Arcos Ldel 2016; Lemstra *et al.* 2012; Moraros *et al.* 2012). Saskatoon, Canada identified substantial health disparities in its low-income neighborhoods compared to its higher income neighborhoods (Lemstra & Neudorf 2008). Similarly, our study found that a majority of the participating STW were residents in the city’s low-income neighborhoods and reported limited employment opportunities, meager financial resources, and lack of adequate and affordable housing. Given these adverse social circumstances, it is not surprising that the use of a validated depression scale in our study indicated that 85.6% of the participating STW were depressed. This is an important finding especially in light of the fact that 38.5% stated the lack of access to mental health therapists was a major concern.

### Strengths and limitations

One of the unique contributions of this study is the fact that the significant independent risk indicators for STW self-reporting a chance of greater than 50% of becoming HIV/AIDS infected were identified. Significant risk factors for HIV infection among the participating STW included being the victim of sexual assault as a child, injecting drugs in the past four weeks, being homeless, and being diagnosed with Chlamydia in the past. Therefore, an effective HIV strategy will be required to take into account and make provisions for mental health services and social supports that directly address these risk factors that make Saskatoon, the city in Canada with the highest rates of new reported cases of HIV (PHAC 2009).

Finally, this study has a number of limitations. It was cross-sectional in nature, which precludes inference of causality. The results were based on self-reported information from STW, who were recruited by using a sample of convenience. Thus, they may be subject to under or over-reporting, social desirability bias, and lack of generalizability. However, previous studies have provided validation of self-reported information among STW populations (Goldstein, Friedman, Neaigus, Jose, Ildefonso & Curtis 1995; Petry 2001). Additionally, given the hard-to-reach nature of STW, it is hoped that further research and community mapping will help ensure that an increased number of STW are accurately identified and successfully recruited to participate in future studies.

## Conclusion

One of the main themes arising from the present study is the need for ownership and community engagement in order to ensure success in the fight to reduce the scourge of HIV. It provides important evidence of the essential sexual and drug-related vulnerabilities associated with HIV infection and offers insight into the design and implementation of effective and culturally sensitive public health intervention and prevention efforts tailored to meet the unique needs of STW in Saskatoon, Canada.

## Recommendations

To be most effective, it is recommended that future HIV intervention and prevention initiatives: (1) use specifically tailored community-based outreach programs for high risk STW, who are drug users, and link them with appropriate drug treatment and HIV/AIDS prevention and treatment services, (2) provide free and confidential, routine HIV counseling and multiple testing opportunities in substance abuse programs, and (3) build capacity among the local, Aboriginal NGOs so as to create culturally sensitive and effective programming that takes into account the drug and HIV-related risk factors prevalent among this vulnerable population.
